# Montreal Veterinary Medical Association

**Published:** 1887-04

**Authors:** Thomas E. Feron

**Affiliations:** Secretary


### MONTREAL VETERINARY MEDICAL ASSOCIATION.

The regular fortnightly meeting of the Montreal Veterinary Medical Association was held in the lecture room of the college on Thursday evening, the 17th inst., at 8 P. M., Professor T. Wesley Mills, second vice-president, in the chair. A case of paralysis in an aged mare was reported by Mr. W. F. Torrence. This elicited from Drs. Mills and McEachran some interesting explanations and comparisons with reference to paralysis in man and animals, Dr. Mills pointing out the fact, that in man it was often due to blood pressure, and he appreciated Dr. McEachran's remarks on its comparative rarity in the lower animals, which he explained might in part be due to the longer retention of tonicity and elasticity in the blood-vessels of animals. In this respect an old dog would compare with a young child.

Mr. C. R. Simpson "Student," read a very interesting paper on "Hog Cholera variously known as Pneumo-Enteritis," "Pig Typhoid" "Swine Plague" etc., etc.,

He said that as yet very little was known of the nature and treatment of the disease, notwithstanding the investigations of very able men on the subject. He believed the disease to be a contagious and infectious eruptive fever, and, it was undoubtedly due to a specific germ. That animals inoculated either with cultures or with exudations direct, presented symptoms identical with those seen clinically.

It was fully as prevalent where hogs run loose in pastures, as where they were confined. Quarantine laws providing for the slaughter of diseased animals and governing the movements of suspected animals, should be rigidly enforced. It was the duty of the inspector, however, to be pretty certain of his diagnosis, as the disease may be compounded with sporadic pneumonia, catarrh, anthrax and common diarrhœa. Inoculations with the exudates seldom failed to produce the disease. While inoculations with the blood of living animals had always failed.

Injection of the virus sometimes produced the disease; in one case where the disease broke out in a drove in Massachusetts, it was found that the owner had fed his hogs on slops into which the rinds of hams had been thrown; these hams came from a part of the country where the disease prevailed extensively. The healthy hogs were isolated and the feeding of the rinds discontinued, when the disease disappeared. Inoculations with attenuated virus had not proved successful ; of a drove of twenty-five so inoculated twenty-one died. The virus used in these inoculations came from M. Pasteur's laboratory. Though animals may recover under medical treatment, they never fatten as well after a severe attack. The reading of the paper was followed by an animated discussion. Dr. Clement, V.S., said that the Germans recognized in what we termed Hog Cholera two distinct diseases, and illustrated the micro-organisms peculiar to each. Dr. McEachran pointed out the great losses occasioned to the farmers in the United States, which, he said, amounted to nearly thirty million dollars annually. He described investigations which had been conducted at this college by Prof. Osler and gave some instructive information on the disease generally. Prof. Mills followed in an admirable address on the importance of scientific training and accurate knowledge on the part of those young men to whom such valuable and important interests were entrusted professionally.

The proceedings were brought to a close with a pathological demonstration by Dr. Clement on the organs of a dog which died from distemper. He illustrated by microscopic preparations the organisms seen in the lungs and spleen.

At the regular meeting, March 3d, 1887, Mr. M. C. Baker, V. S., president, in the chair, Mr. Thomas E. Feron, student, reported a case of laminitis in a horse.

An interesting paper was read by Mr. Frank H. Miller, student, on indigestion in the horse. He stated as his opinion that insufficient pancreatic secretion was a very common cause of chronic indigestion, and quoted from Strangeway, McFayden and Foster, to support his theory. He considered flatulent colic as much more serious than the spasmodic form, both on account of the liability to rupture and because as he believed it was much more liable to be followed by enteritis. He favored the early use of the trochar, where there was much tympanitis. An animated discussion followed.

THOMAS E. FERON,
Secretary

